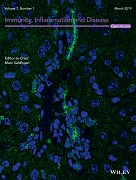# Cover

**DOI:** 10.1002/iid3.247

**Published:** 2019-03-14

**Authors:** Kamal U. Saikh, Jennifer L. Dankmeyer, Xiankun Zeng, Robert G. Ulrich, Kei Amemiya

**Affiliations:** ^1^ Department of Immunology Army Medical Research Institute of Infectious Diseases Frederick Maryland USA; ^2^ Department of Bacteriology Army Medical Research Institute of Infectious Diseases Frederick Maryland USA; ^3^ Department of Pathology Army Medical Research Institute of Infectious Diseases Frederick Maryland USA

## Abstract

The cover image is based on the Original Research *An increase in intracellular p62/NBR1 and persistence of Burkholderia mallei and B. pseudomallei in infected mice linked to autophagy deficiency* by Kamal U. Saikh et al., DOI: 10.1002/iid3.239.